# Fatal systemic AA amyloidosis with pulmonary *Mycobacterium avium* infection: an autopsy case report

**DOI:** 10.1128/asmcr.00193-25

**Published:** 2026-03-04

**Authors:** Yui Takahashi, Shigeo Hanada, Hironori Uruga, Takayuki Tsujimoto, Eiyu Tsuboi, Hiroshi Nakahama, Yuichiro Nei, Takahiro Mitsumura, Atsushi Miyamoto, Meiyo Tamaoka

**Affiliations:** 1 Department of Respiratory Medicine, Respiratory Center, Toranomon Hospital 13600, Tokyo, Japan; 2 Okinaka Memorial Institute for Medical Research 545161, Tokyo, Japan; 3 Department of Pathology, Toranomon Hospital 13600, Tokyo, Japan; Vanderbilt University Medical Center, Nashville, Tennessee, USA

**Keywords:** AA amyloidosis, autopsy, gastrointestinal amyloidosis, *Mycobacterium avium*, pulmonary infection, nontuberculous mycobacteria

## Abstract

**Background:**

Occurrences of amyloid A (AA) amyloidosis secondary to nontuberculous mycobacteria (NTM) are rare. Here, we report a fatal case of AA amyloidosis in a woman with pulmonary *Mycobacterium avium* infection, with autopsy confirming extensive amyloid deposition across multiple organs.

**Case Summary:**

A 59-year-old woman was diagnosed with pulmonary *M. avium* infection in 2018 but discontinued follow-up. In March 2022, she re-presented with disease progression and commenced standard oral therapy with daily clarithromycin, rifampicin, and ethambutol. After 12 months of therapy, her regimen was discontinued for 3 months due to persistent diarrhea. Upon resuming treatment, progressive weight loss persisted, leading to hospitalization in July 2023. On admission, she was severely underweight. Imaging revealed bilateral lung infiltrates with cavitary lesions. Laboratory evaluation showed elevated inflammatory markers, hypoalbuminemia, and sputum positive for acid-fast bacilli on smear and positive for *M. avium* by transcription–reverse transcription concerted reaction and culture. Gastrointestinal biopsies showed AA amyloid deposits confirmed by direct fast scarlet staining and immunohistochemistry. Despite modified antibiotic regimens, her condition deteriorated, leading to CO₂ narcosis, multiorgan failure, and ultimately death at 123 days after admission. Autopsy confirmed extensive amyloid deposition and advanced pulmonary mycobacterial disease.

**Conclusion:**

This case highlights a rare but severe presentation of systemic AA amyloidosis occurring with pulmonary *M. avium* infection, suggesting a possible association between uncontrolled *M. avium* infection and systemic AA amyloidosis development. In individuals with pulmonary NTM presenting with refractory diarrhea and weight loss, clinicians should consider AA amyloidosis and pursue appropriate biopsy investigations.

## INTRODUCTION

Nontuberculous mycobacteria (NTM) comprise over 190 *Mycobacterium* species, excluding the *Mycobacterium tuberculosis* complex, *Mycobacterium leprae*, and *Mycobacterium ulcerans* (1). NTM can infect various anatomical sites, most frequently involving the lung (2, 3). The majority of pulmonary NTM infections are accounted for by *Mycobacterium avium* complex (MAC) (1), typically presenting with cough, sputum production, fatigue, weight loss, and low-grade fever. Although the disease often progresses slowly, some patients experience worsening symptoms and poor outcomes despite treatment (4).

Amyloidosis results from extracellular deposition of insoluble amyloid fibrils and is classified as localized or systemic based on tissue involvement. The amyloid type is determined by the precursor protein: transthyretin-derived amyloid (ATTR), immunoglobulin light chain-derived amyloid (AL), and serum amyloid A (SAA)-derived amyloid (AA). AA amyloidosis most commonly arises secondary to chronic inflammatory conditions, such as rheumatoid arthritis (RA), inflammatory bowel disease, or tuberculosis (TB) (5, 6). AA amyloidosis secondary to NTM infection is rare. Herein, we report a case of systemic AA amyloidosis associated with pulmonary *M. avium* infection, with autopsy confirming extensive amyloid deposition across multiple organ systems.

## CASE PRESENTATION

**Fig 1:**
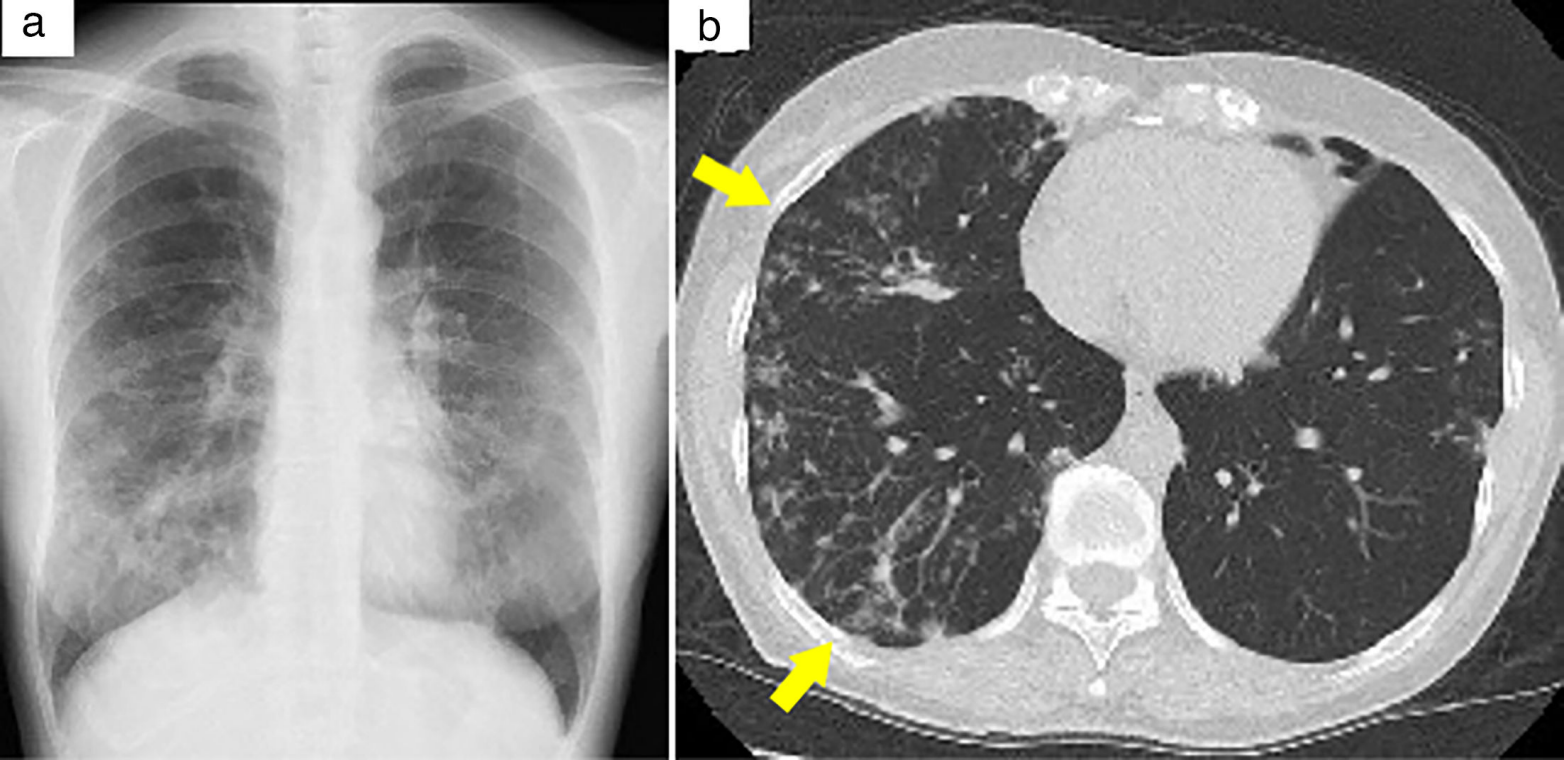
Chest radiography and high-resolution computed tomography (HRCT) at initial visit. (**a**) Chest radiograph and (**b**) HRCT image showing small centrilobular nodules, patchy consolidation, and bronchiectasis, suggesting a nodular bronchiectatic type pulmonary nontuberculous mycobacterial disease (arrows).

A 59-year-old woman with no significant medical history presented to our hospital in January 2018 owing to an abnormal shadow on her chest radiograph. Chest high-resolution computed tomography (HRCT) revealed centrilobular nodules, patchy consolidation, bronchiectasis, and bronchial wall thickening (Fig. 1). The patient tested positive for anti-glycopeptidolipid-core immunoglobulin A antibodies. Bronchial wash fluid was collected for acid-fast bacillus (AFB) smear, transcription–reverse transcription concerted (TRC) assay, and culture. The AFB smear test was positive. A nucleic acid amplification test using the TRC reaction method (TRC Rapid, Tosoh Corporation, Tokyo, Japan) detected *M. avium* and was negative for *Mycobacterium intracellulare*. Cultures were performed on liquid media (MGIT 960, Becton Dickinson, Sparks, MD, USA). Following growth, species identification by matrix-assisted laser desorption/ionization (MALDI) time-of-flight mass spectrometry with the MALDI Biotyper system (Bruker Daltonics, Bremen, Germany) confirmed *M. avium*. These microbiological findings fulfilled the diagnostic criteria for NTM pulmonary disease according to the American Thoracic Society/Infectious Diseases Society of America (ATS/IDSA) guidelines; however, the patient discontinued follow-up. In March 2022, she re-presented with disease progression, showing cavitary lesions and pleural thickening on HRCT. Based on the ATS/IDSA statement, daily therapy with clarithromycin (800 mg/day), ethambutol (500 mg/day), and rifampicin (450 mg/day) was initiated. After 12 months of therapy (March 2023), persistent diarrhea led to discontinuation of therapy. Suspecting drug-induced diarrhea, treatment was resumed in June 2023 with intestinal and antidiarrheal support. However, her symptoms persisted, causing a 10 kg weight loss over 6 months and ultimately necessitating hospitalization in July 2023.

**Fig 2:**
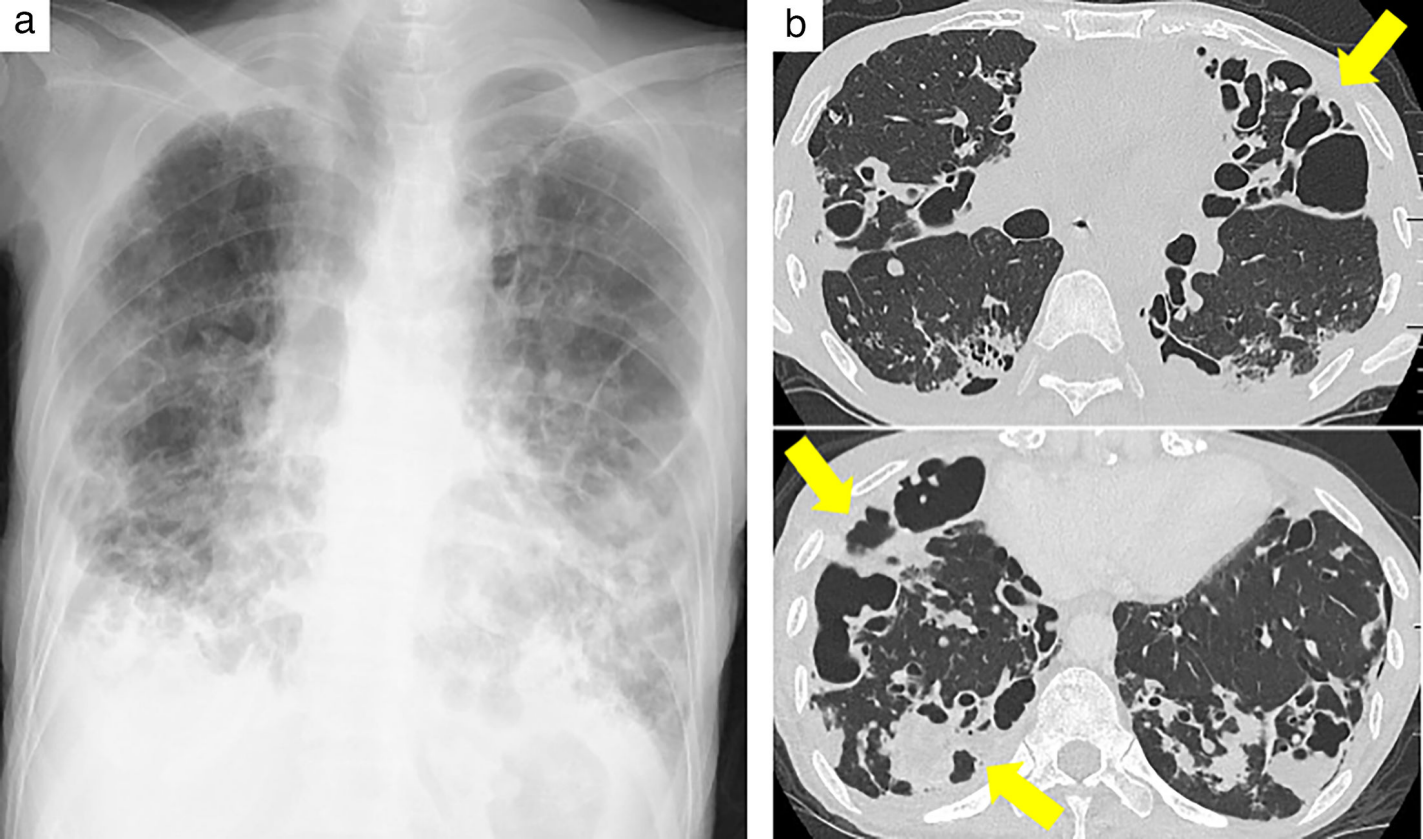
Chest radiography and high-resolution computed tomography (HRCT) on admission. (**a**) Chest radiograph showing infiltrates, reticulonodular opacities, and cavities in both lungs. (**b**) HRCT image showing bronchiectasis, multiple nodules, consolidations, pleural thickening, and cavity lesions of various sizes, with a bilateral and multilobular distribution. Findings indicate marked disease progression (arrows).

**Fig 3:**
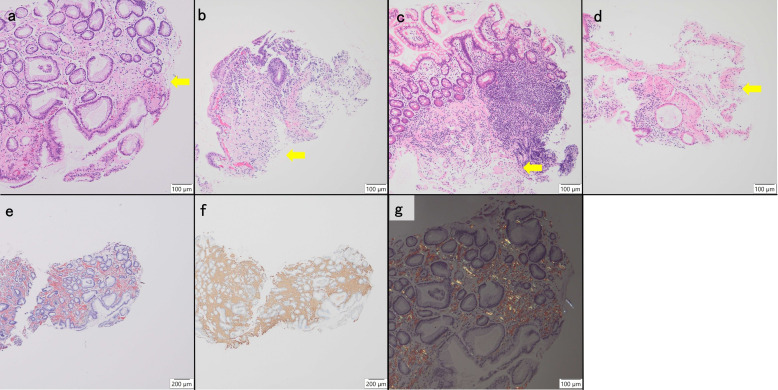
Amyloid deposition in the gastrointestinal tract. Amyloid deposition seen as pale eosinophilic amorphous material in the lamina propria and muscularis mucosa of (**a**) the stomach, (**b**) duodenum, (**c**) ileum, and (**d**) colon (arrows). Amyloid is positive for direct fast scarlet staining (**e**) and AA-type on immunohistochemistry with Anti-Human Amyloid A (clone mc1) (Dako) (**f**). Green birefringence is observed in the amyloid deposits by brief polarization (**g**).

Upon admission, the patient’s weight was 36 kg (body mass index [BMI], 14 kg/m^2^). She presented with bilateral coarse crackles and rhonchi on chest auscultation. Vital signs indicated hypotension (96/75 mmHg), a heart rate of 93 beats/min, tachypnea (24 breaths/min), and oxygen saturation of 97% on 1 L/min nasal oxygen. Chest radiographs revealed bilateral infiltrates, reticulonodular opacities, and cavities. Arterial blood gas on room air showed mild hypoxemia (PaO_2_, 78 mmHg) and hypercapnia (PaCO_2_, 49 mmHg). Laboratory tests demonstrated elevated white blood cell count (15,900/μL; normal range, 3,300–8,600/μL), high C-reactive protein (CRP) (11.29 mg/dL; normal range, < 0.14 mg/dL), hypoalbuminemia (1.1 g/dL; normal range, 4.1–5.1 g/dL), and low total protein (5.1 g/dL; normal range, 6.6–8.1 g/dL) levels, indicating significant inflammation and malnutrition. Sputum testing was positive for AFB on smear, and *M. avium* was confirmed by TRC assay and culture. Drug susceptibility testing demonstrated susceptibility to clarithromycin and amikacin, with minimum inhibitory concentrations of 0.12 and 2μg/mL, respectively. Chest HRCT revealed marked disease progression, with bronchiectasis, multiple nodules, consolidations, pleural thickening, and variable-sized cavities across multiple lobes bilaterally (Fig. 2). After admission, the patient underwent esophagogastroduodenoscopy (EGD) and colonoscopy for persistent diarrhea and weight loss. EGD revealed gastric ulcers and friable duodenal mucosa, while colonoscopy showed inflammation extending from the ileum to the ascending colon. Biopsies from the stomach, duodenum, and colon demonstrated amyloid deposition, confirmed by direct fast scarlet staining and immunohistochemistry using anti-human amyloid A (clone mc1, Dako, Glostrup, Denmark), indicating AA-type amyloidosis (Fig. 3). A diagnosis of systemic AA amyloidosis was established based on these biopsy findings. Laboratory evaluation revealed an elevated SAA level of 52 mg/L (normal range, <3.0 mg/L) and a CRP level of 2.64 mg/dL. Given the absence of other chronic inflammatory conditions, the longstanding pulmonary NTM infection was considered the most likely underlying cause of AA amyloidosis. Management primarily focused on controlling the underlying infection. Based on ATS/IDSA guidelines and antimicrobial susceptibility testing, initial therapy with oral clarithromycin, ethambutol, and rifampicin was continued. However, due to suspected poor absorption from severe diarrhea, therapy was modified to daily intravenous azithromycin (250 mg/day), intramuscular streptomycin (500 mg/day) 3 days per week, and daily oral ethambutol (500 mg/day). At 1 month after admission, CO₂ narcosis (PaCO₂, 90 mmHg) necessitated noninvasive positive pressure ventilation, and intravenous piperacillin-tazobactam (4.5 g every 8 h, 13.5 g/day) was added. Despite treatment for NTM and antibiotics for fever and purulent sputum, her respiratory and nutritional status worsened, with her weight declining to 28 kg (BMI, 10.9 kg/m^2^). Although a bone marrow biopsy was not performed due to respiratory instability, blood and urine cultures were negative; thus, disseminated NTM was not microbiologically confirmed. She developed multiorgan failure and died 123 days after admission. Her SAA concentration had increased markedly from 52 at the time of AA amyloidosis diagnosis to 259.6 mg/L at 1 month prior to death (CRP was 5.66 mg/dL).

**Fig 4:**
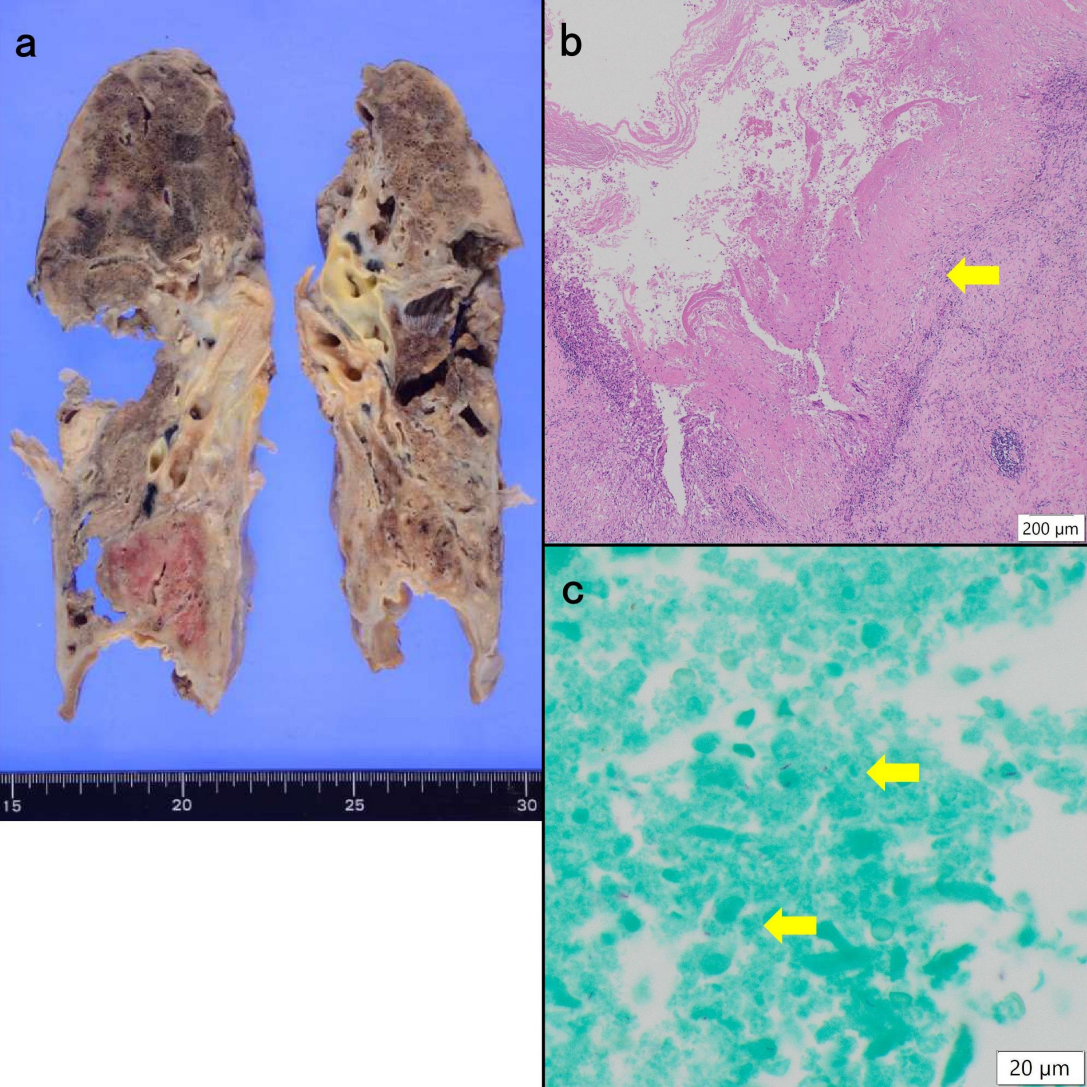
Autopsy findings of the lung lesions. Gross findings on autopsy show calcified, cavitated lesions, and caseous nodules in both lungs (**a**). Microscopically, extensive caseous necrosis is evident (**b**). Ziehl-Neelsen staining reveals red-stained bacilli (**c**) (arrows).

**Fig 5:**
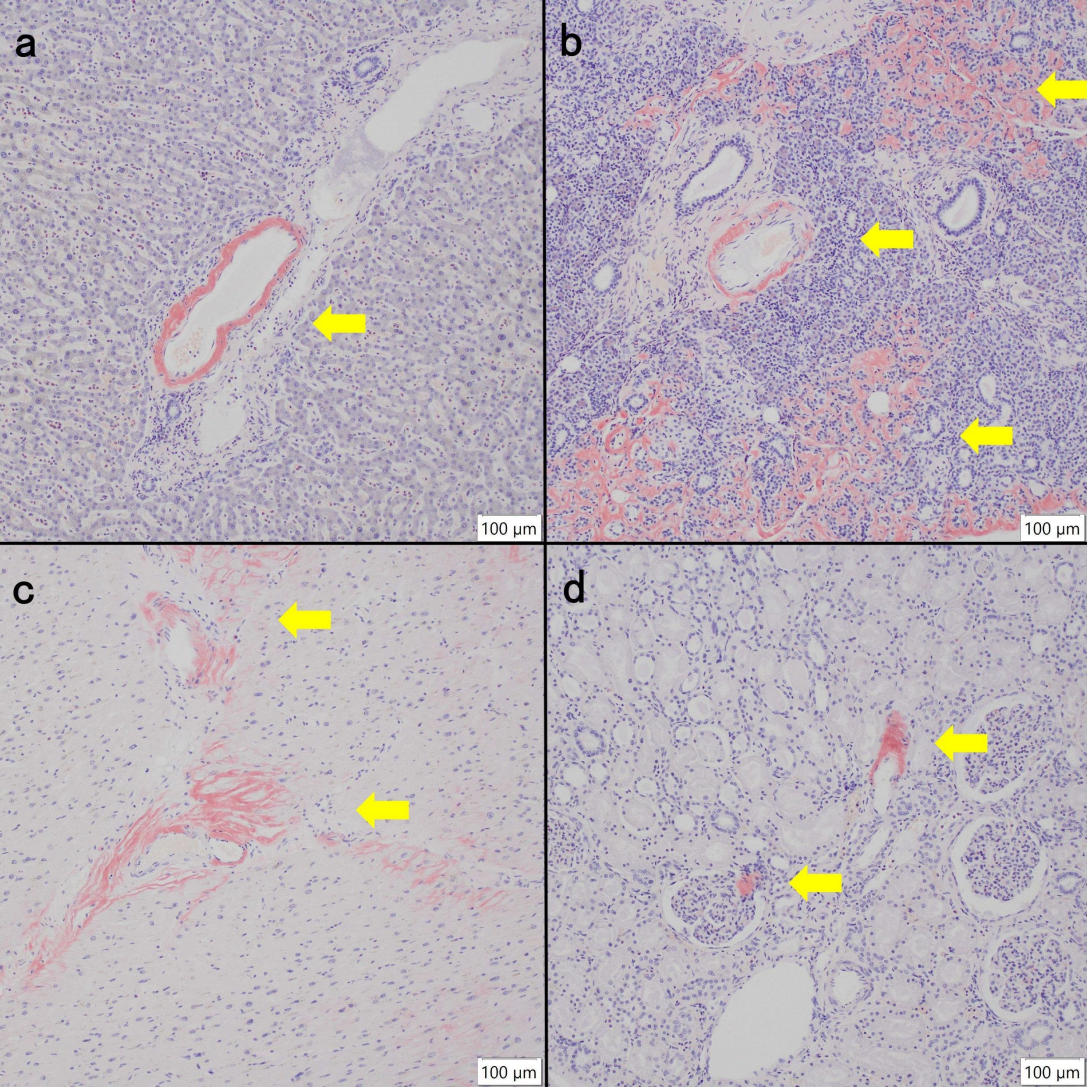
Amyloid deposition in visceral organs. On direct fast scarlet staining, amyloid is positive in (**a**) liver (hepatic artery wall), (**b**) pancreas (blood vessel wall and stroma), (**c**) heart (cardiac muscle, blood vessel wall, and stroma), and (**d**) kidney (intraglomerulus and stroma) (arrows).

An autopsy was performed. Gross examination revealed cavities with caseous nodules and calcifications, bronchiectasis, and pleural thickening in both lungs (Fig. 4). Microscopically, epithelioid granulomas with caseous necrosis, collapsed fibrosis, and organized fibrosis were observed. Membranous bronchi were associated with traction bronchiectasis. Noncaseating granulomas were identified throughout the bronchial walls and alveolar regions. Though AFB stains and cultures were not performed, granulomas were not detected in any other autopsy organs. No disseminated NTM infection was confirmed. Amyloid deposition was detected in multiple organs, including the digestive tract (esophagus, stomach, duodenum, small intestine, and colon), heart, kidney, liver, spleen, lung, pancreas, thyroid, parathyroid gland, submandibular gland, and skin (Fig. 5). Immunohistochemically, the amyloid was positive for AA and negative for amyloid light chain-kappa, amyloid light chain-lambda, beta 2-microglobulin, and ATTR.

## DISCUSSION

AA amyloidosis results from the misfolding and aggregation of SAA protein into insoluble fibrils with a β-sheet structure (6, 7). It typically arises secondary to chronic inflammatory conditions. Although historically associated with TB, the most current cases occur in RA, which accounts for 60%–70% of cases (5, 6, 8). Reported mean survival for AA amyloidosis is approximately 24 months (5). Pulmonary NTM are a rare underlying cause, with only four cases described to date (9–12) (Table 1). As available evidence is limited, conclusions regarding prognosis should be interpreted with caution. In the present case, the patient died 4 months after the diagnosis of AA amyloidosis. Poor prognostic indicators include hypoalbuminemia (<2.5 g/dL), end-stage renal failure at treatment initiation, and elevated SAA levels during the course of the disease (5, 6). The risk of death increases significantly when the SAA levels are ≥45 mg/L, especially if the levels exceed 155 mg/L (6). The patient in this case had severe hypoalbuminemia (1.1 g/dL) and a rising SAA level, reaching 259.6 mg/L; both signify a poor prognosis.

**TABLE 1:** Reported cases of AA amyloidosis in association with pulmonary nontuberculous mycobacterial infection^*a*^

Case	Age (yr)	Sex	Mycobacteria	Time from NTM diagnosis	Symptoms/signs	Outcome
1 (9)	73	M	*Mycobacterium simiae*	2 years 6 months	Epigastric pain/weight loss	Death (unknown)
2 (10)	75	M	*M. intracellulare*	6 years 5 months	Dyspnea/edema	Death (14 months)
3 (11)	64	F	*Mycobacterium abscessus*	10 months	Diarrhea/abdominal pain	Death (4 months)
4 (12)	75	F	*M. avium*	2 years	Diarrhea/abdominal pain	Resolved
5 (present case)	59	F	*M. avium*	5 years	Diarrhea/weight loss	Death (4 months)

^*a*^ AA, amyloid A; NTM, nontuberculous mycobacterium; M, male; F, female.

Management of AA amyloidosis targets the underlying inflammatory disease (4). Anti-human interleukin-6 (IL-6) receptor antibodies can reduce SAA and improve outcomes in noninfectious etiologies, particularly RA (13). However, its efficacy in infection-associated AA amyloidosis remains controversial since immunosuppressive therapy may exacerbate infections. In the present case, anti-IL-6 therapy was withheld due to poorly controlled *M. avium* infection, and treatment of the underlying NTM infection was prioritized. Had AA amyloidosis been recognized earlier, more aggressive nutritional support, earlier initiation of parenteral antibiotic therapy, or consideration of anti-IL-6 therapy after adequate infection control might have been possible.

The incidence of pulmonary NTM disease in Japan has increased significantly over time. According to a nationwide hospital-based survey, the incidence rate in 2014 was approximately 14.7 cases per 100,000 person-years, which represents an increase of approximately 2.6-fold compared to the incidence rate reported in 2007 (14). Pulmonary NTM disease, including MAC and other NTM species, presents as either fibrocavitary (FC) or nodular bronchiectatic (NB) forms. The FC type progresses rapidly with a poorer prognosis and a lower 5-year survival rate (approximately 60%) compared with that of the NB form (>90%) (15). While the NB type may be monitored and some cases improve spontaneously without treatment, early treatment is advised for FC or extensive disease.

Our patient initially had NB-type disease but progressed to extensive FC-type with lung destruction. She had no identifiable immunosuppressive conditions, including HIV infection or corticosteroid use, and no clinical features suggestive of congenital immunodeficiency. In addition, there was no underlying structural lung disease that could account for the severity of the NTM infection. Autopsy confirmed widespread amyloid deposits and cavitary lesions with caseous necrosis, suggesting that poorly controlled *M. avium* infection contributed to inadequate management of AA amyloidosis. Diarrhea and weight loss during NTM treatment may result from drug-related effects, gastrointestinal NTM involvement, or, rarely, AA amyloidosis. As diagnosis requires specific staining, it is often overlooked. When symptoms persist, a biopsy with amyloid staining should be considered to detect underlying amyloidosis.

This case highlights a rare and fatal instance of systemic AA amyloidosis associated with pulmonary *M. avium* infection. Although causation cannot be definitively established, the temporal progression, ongoing inflammation, and rising SAA levels support a correlation. Despite initial antimicrobial therapy, disease progression resulted in severe malnutrition, type 2 respiratory failure, and multiorgan dysfunction. Autopsy confirmed widespread amyloid deposition across multiple organs. The case emphasizes the need to consider AA amyloidosis in individuals with refractory gastrointestinal symptoms and weight loss during NTM treatment. Early biopsy and amyloid staining are essential for diagnosis.
